# Communicating climate change findings from IPCC reports: insights from outreach events in India

**DOI:** 10.1007/s10584-021-03224-8

**Published:** 2021-10-22

**Authors:** Minal Pathak, Joyashree Roy, Shaurya Patel, Shreya Some, Purvi Vyas, Nandini Das, Priyadarshi Shukla

**Affiliations:** 1 Technical Support Unit (TSU), Working Group III (WGIII), Intergovernmental Panel On Climate Change (IPCC), Geneva, Switzerland; 2 Global Centre for Environment and Energy, Ahmedabad University, Ahmedabad, Gujarat India; 3 Asian Institute of Technology, Klong Luang, Pathum Thani, Thailand; 4 Jadavpur University, West Bengal Kolkata, India; 5 Global Change Programme, Jadavpur University, Kolkata, West Bengal India; 6 WGIII, IPCC, Geneva, Switzerland; 7 Amrut Mody School of Management and Global Centre for Environment and Energy, Ahmedabad University, Ahmedabad, Gujarat India

**Keywords:** Public engagement, IPCC, Communication, Outreach, India

## Abstract

In recent years, the Intergovernmental Panel on Climate Change (IPCC) has been collaborating with Indian institutions to organise outreach events. This essay draws on the perspective of participants, speakers and organisers of 17 in-person outreach events conducted across India in 2018 and 2020, to share insights and recommendations for future IPCC events in India and other developing country contexts. The formats analysed in this essay range from panel events with very large public audiences to more focused workshops, meetings and seminars. Target audiences covered both academic and non-academic audiences and included researchers, teachers, students, industry and NGOs. The events, while achieving their main objective of communicating the findings of IPCC reports, also provided a platform for open discussion of localised climate impacts and good practices in adaptation and mitigation. There are, however, notable challenges to public outreach in India, specifically in terms of attracting an adequate number of participants, experts’ availability, communicating to a diverse audience and translation into local languages. The biggest challenge faced by speakers was a lack of knowledge about the number of attendees and the composition of the audience prior to an event. It is our recommendation that future outreach events in India are designed to be interactive, tailored to the regional context and complemented by simplified communication materials. Speakers should be provided with audience information and background prior to the event, and greater reach into rural areas, including school children, could be achieved with material in local languages. Additionally, event organisers often require logistical and operational support to host outreach events.

## The communication of IPCC reports in India

The IPCC’s Assessment Reports are widely regarded as among the most credible sources of peer-reviewed evidence on climate change, summarising the state of knowledge about the physical science, its potential impacts and options to mitigate the greenhouse gas emissions that drive it (Howarth and Painter 2016). The importance of communicating climate change for building public awareness and engagement and the inherent challenges in doing so have been extensively discussed in the literature (Moser 2010; Patt and Weber 2014; Rapley et al. 2014). In its early years, the IPCC did not prioritise raising the topic of climate change in the public consciousness (Lynn 2018). Arguably, this changed with the publication of the Fourth Assessment Report (AR4) in 2007 (IPCC 2007), which attracted widespread attention around the world and led to the IPCC winning the Nobel Peace Prize in 2007 (Norwegian Nobel Committee 2007).

Climate change is a complex and interdisciplinary topic, making presentation challenging particularly to diverse groups (Rapley et al. 2014; Suldovsky 2017; Howarth et al. 2020; Cook and Overpeck 2019; van Swol et al. 2021). IPCC reports are produced by an iterative process: successive drafts are revised several times by authors in response to comments from a formal review process involving governments and expert reviewers (Howarth and Painter 2016; Amelung et al. 2016). Authors respond to thousands of review comments over the course of an assessment cycle, making it a truly comprehensive scientific assessment. However, findings are often condensed into highly complex graphs, which may be inaccessible for non-specialist audiences (Amelung et al. 2016; Harold 2017; Van Den Broek 2021) or likely to be misinterpreted (Fischer et al. 2020; McMahon et al. 2015). The IPCC faces particular challenges, especially in terms of communicating complexity and uncertainty (Howe et al. 2019; Ho and Budescu 2019; Budescu et al. 2014; Mehta et al. 2019; Bajcinovci and Jerliu 2016).

Communication of IPCC reports has improved significantly over the years as a result of efforts to present key findings in a more accessible way, while still maintaining scientific integrity. For example, developments in the most recent assessment reports include communications training for IPCC authors, press releases with inputs and engagement from the working group co-chairs, dedicated communications staff within each working group and direct contact between the media and IPCC authors. Developing accessible communication materials, including standard slide decks and short videos in which IPCC authors and co-chairs clearly and concisely communicated the key findings from the reports, has been received extremely well (IPCC 2016).

Public understanding and attitudes towards climate change in India have also evolved considerably in recent years. A national survey of 4031 Indian adults showed people had personally observed changes in local rainfall, temperatures and weather patterns and linked an improvement in the perception and understanding of climate change to an increased focus on the topic in the mainstream media (Leiserowitz and Thaker 2012). According to the Global Trustworthiness Index survey conducted in 2019, two-thirds of India’s urban population found scientists to be the most trusted source for information on climate change (IPSOS 2019). An audience segmentation analysis of the Indian population showed a fifth were well-informed of climate risks and supportive of national actions, 24% were convinced that climate change is happening and is a serious problem, while more than half were either undecided, unconcerned, indifferent or completely disengaged (Leiserowitz et al. 2013).

The publication of the IPCC’s Fourth Assessment Report (AR4) in 2007 marked the beginning of a more systematic effort to engage a wide range of Indian society with the key messages of IPCC reports. During dedicated outreach events, IPCC authors directly engaged with a variety of stakeholders. These initiatives were realised through a partnership between the Working Group III Technical Support Unit (including the in-house communications team) and the institutional and subnational government connections of IPCC authors. As well as engaging the media and policymakers with IPCC findings, these outreach events were intended to provide a platform to listen to a diverse range of voices, including state and non-state actors, about adaptation and mitigation initiatives at local, sectoral, subnational and regional levels. Furthermore, these outreach events were intended to create a dialogue with the public and to satisfy a growing appetite among various social groups for reliable scientific information about the science of climate change and the solutions for addressing it.

However, with the exception of the occasional event proceedings, no previous study has assessed the extent to which IPCC outreach events in India have been successful in achieving their aims. Neither has there been any formal documentation of the specific challenges and barriers to organising such events in India. To the best of our knowledge, this essay is the first attempt to do so and, as such, we hope the findings will be useful not only for future IPCC outreach events in India but also in other developing country contexts.

## A preliminary analysis of outreach events in India (2018–2020)

As part of the current Sixth Assessment Report (AR6) cycle, the IPCC has published three special reports: Global Warming of 1.5 °C (SR15), Climate Change and Land (SRCCL) and the Ocean and Cryosphere in a Changing Climate (SROCC). Disseminating the findings from these special reports has been the primary objective of outreach events organised in India in recent years. Table 1 summarises observations from 17 official in-person outreach events organised by the IPCC communications team and the Working Group III Technical Support Unit between 2018 and 2020. The following subsections present an initial analysis of survey responses collected from 31 individuals involved in these outreach events (21 participants, 6 speakers and 4 organisers, including the authors of this essay). Participants were asked to recall their experience of the events they had attended through a questionnaire administered using Google Forms. Insights from separate outreach events conducted for university and school students from various grades starting from middle level to high school level in three Indian cities — Ahmedabad, Kolkata and Chennai — are also documented.

**Table 1:** List of 17 in-person outreach events in India in 2018–2020 analysed in this essay

No	Title of the event	Date and location	Purpose	Type of event	Audience	Duration	Number of participants
1	Roundtable on Urban India and Climate Change Research	December 2018, New Delhi	Linked to SR15 findings — presented on urban transformations for 1.5 °C: A revised framework for Indian cities	Roundtable	Researchers	Half day	70
2	Road to IPCC’s Sixth Assessment Report	January 2019, New Delhi	Share findings of the IPCC special report SR15 C and AR6 report — foster engagement across different sectors and stakeholders acting on climate change	Conference, 5 sessions	Policymakers, industry (steel, cement, automobile), finance sector, civil society organisations	Full day	150
3	Systems Transformations for Global Warming of 1.5 °C	January 2019, Ahmedabad	Share findings of the IPCC special report SR15 and AR6 report — foster engagement across different sectors and stakeholders acting on climate change	Conference, 4 sessions	Policymakers, researchers, university students, civil society organisations	Full day	83
4	One Planet: Re-imagining our future	January 2019, Ahmedabad	Share findings from IPCCs special report on global warming of 1.5 °C	Talk/lecture, 1 session	School students	Half day	100
5	Climate Change and India: Conversing Across Disciplines	March 2019, New Delhi	Share findings of the special reports — SR15 and SRCCL	Panel discussion	General public	Half day	NA
6	Climate Change and Industry: Towards a Low Carbon Transformation	July 2019, Ahmedabad	Discuss the methodology of reporting CO_2_ emissions — shared findings of the SR15 and SRCCL	Workshop	Industry representatives	Full day	50
7	Conference on Climate Change and Land	August 2019, Ahmedabad	Share findings of the special report — SRCCL	Conference, 4 sessions	Policymakers, researchers, students, civil society organisations	Full day	70
8	UNCCD COP 14 — Introducing the IPCC Special Report on Climate Change	September 2019, New Delhi	Share findings of special report — SRCCL	Panel discussion (science day), 1 session	Policymakers, civil society organisations	Half day	NA
9	The Work and Findings of the Intergovernmental Panel on Climate Change: Three special reports	October 2019, Hyderabad	Share findings of the three special reports (SR15, SRCCL, SROCC) and IPCC process and scope	Plenary Session during the Tenth Biennial Conference of the Indian society for ecological economics (INSEE)	International participants: researchers, subject experts, early career research scholars, students, university faculty, university students	Special IPCC panel	150
10	The Work and Findings of the Intergovernmental Panel on Climate Change: Three special reports	October 2019, Hyderabad	Share findings of the three special reports (SR15, SRCCL, SROCC) and IPCC process and scope	Workshop at EPTRI	With researchers from trainers from Environment Protection and Training Research Institute (EPTRI)	Half day	20
11	The Work and Findings of the Intergovernmental Panel on Climate Change: Three special reports	October 2019, Hyderabad	Share findings of the three special reports (SR15, SRCCL, SROCC) and IPCC process and scope	Workshop at International Crops Research Institute for the Semi-Arid Tropics (ICRISAT), Hyderabad	Subject experts, researchers at ICRISAT	Half day	60
12	The Work and Findings of the Intergovernmental Panel on Climate Change: Three special reports	October 2019, Hyderabad	Share findings of the three special reports (SR15, SRCCL, SROCC) and IPCC process and scope	NGO, Centre for World Solidarity (CWS) and Centre for People’s Forestry	Multiple NGO groups, leaders, action researchers, field workers, farmer association representatives	Half day	25
13	IPCC Dissemination Workshop: Key Messages from Three Special Reports of IPCC (SR 1.5, SRCCL and SROCC)	November 2019, Kolkata	Share findings of the three special reports (SR15, SRCCL, SROCC) and IPCC process and scope	Large public events in well-equipped state of the art auditorium	Policymakers, representative of state government, civil society, university/college faculty, researcher, formalist, students, school students, teachers, media persons	Two full days	600
14	Roundtable Conference on Cities and Climate Change	December 2019, Ahmedabad	Share findings of special reports — SR15 and SRCCL	Roundtable, 4 sessions	Policymakers, researchers, students, civil society organisations	Full day	65
15	Mitigation Landscape in a Carbon Constrained World	December 2019, Ahmedabad	Share findings of special reports — SR15 and SRCCL and how they are linked to mitigation	Workshop, 3	Researchers, Academics, Experts, Students and Social Workers	Full day	45
16	Climate in the Age of Anthropocene — Kolkata Literary Meet	January 2020, Kolkata		Conversation/panel discussion	General public	Half day	
17	Sustainability Fair — Indian Institute of Technology Gandhinagar	February 2020, Gandhinagar	Dissemination of all 3 special reports of IPCC	Fair and conference	Students, Researchers, Academics, Scientist, Industry, NGOs, CBOs	Full day	200

### Insights from the participants’ survey

Participants of the 17 formal IPCC outreach events listed in Table 1 were asked why they chose to attend the event in question. More than 70% said that they were familiar with the IPCC special reports and wanted to know more about these reports directly from authors. Nearly a half (47.6%) were from academia, 14.3% were experts, policymakers or from NGOs, with the remaining participants from private business/organisations or independent consultant/freelancers. About a fifth of the participants (19%), though not familiar with the IPCC reports per se, heard about the event and thought it might be helpful for their own work. The remaining participants attended following extremely positive experiences at previous events hosted by the IPCC authors and co-organisers.

Among the aspects that participants enjoyed most about the outreach events they had attended were the speakers’ expertise and background,^1^ their communication skills^2^ and the quality of the presentations. Some respondents commented on the smooth organisation of the events, the representation from a wide range of stakeholders^3^ and the diversity of discussion.^4^ Respondents also appreciated that the selection of speakers reflected a diverse range of perspectives.^5^

Two-thirds of participants said they had established new contacts at the outreach event that they attended. A majority of the participants (90%) felt that there was enough time for the discussions during the event and felt that the speakers were able to connect with wider public values or points of local interest. None of the respondents found English language to be a barrier for participation (though it is important to note that all the respondents came from urban areas, which suggests they were likely to be comfortable with English language). Greater reach into rural areas and to school students who study in local languages could be achieved with material in local languages (Table 2).

Participants were asked for their views on different presentation formats within the event they attended, covering (i) short sessions on individual topics; (ii) panel discussions; (iii) open Q&A and (iv) a mix of all the above. Most participants found open Q&A sessions to be the most effective form of communication, followed by ‘short sessions on individual topics’. Most of the participants felt that the answers to the questions raised by the audience were dealt with appropriately by the speakers.^6^ One way communication through panel discussions was the least preferred format. Sustainable practices including local delicacies for lunch and refreshments and an eco-friendly conference bag were appreciated by participants.^7^

Eighty percent of participants felt they better understood the urgency of the climate actions required to limit global warming to 1.5 °C after attending an IPCC outreach event. Additionally, 71.4% of respondents felt positive about taking climate action after the event; 23.8% of responses were neutral. More than half of respondents felt their knowledge had increased as a result of the event, particularly towards certain specific sectors. For example, one respondent found his knowledge towards climate mitigation had improved.^8^ Another felt better able to connect related issues such as air pollution, health, land use and food supply chains to climate change.^9^ In other cases, participants commented that insights shared during the event were relatable to their professional and personal life, i.e. that they were able to relate the information to choices about energy consumption, waste, food, transportation and adaptation during heat extremes. One respondent spoke about how such outreach events have established themselves as platforms where “climate discussions are expressible, healthy and open” (Table 2).^10^

**Table 2:**
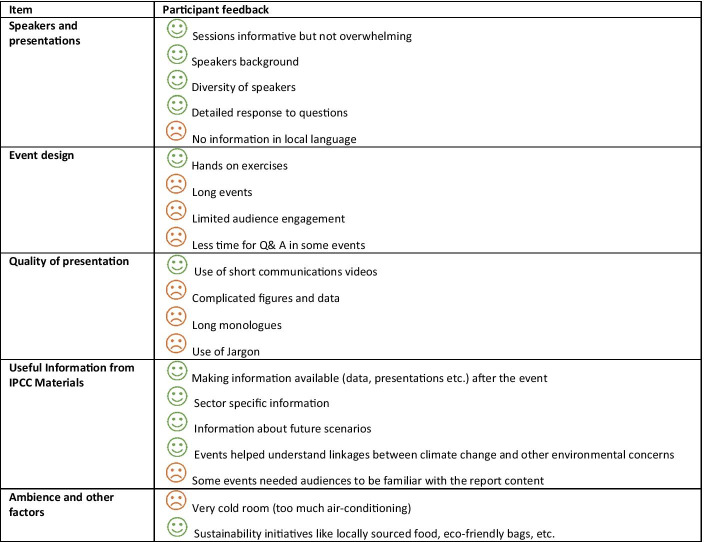
Summary of participants’ responses

#### What could be improved?

A few participants commented that the presentations could focus more on the socio-economic and opportunity costs imposed by addressing climate change for particular regions. One respondent expressed a dislike for the sector specific discussion. One possible reason might be their personal disciplinary bias, topic preferences and interest (in this specific case, the discussion was about species and ecosystems). Two respondents highlighted specifics about the event venue that they disliked, including the small room size and thermal discomfort.

One respondent expressed a view that there were too many speakers, which made the event feel lengthy. Another mentioned a desire to have more tangible outcomes such as discussion papers and requested that speakers’ presentations be made available to the participants after the event.^11^ A few respondents highlighted limited interaction between speakers and the audience during the event.^12^ Some participants felt some of the topics presented required prior knowledge or familiarity with the IPCC reports. Similarly, some respondents found complex visuals and excessive use of technical terms discouraging. Including audio-visuals instead of relying only on infographics helped understand certain topics more clearly. For example, at one of the events organised in Kolkata, a video prepared by the IPCC communication team was very well received. Based on the authors’ observations, not all the outreach events analysed here were inclusive for people with physical challenges and sensory impairments. Therefore, making climate change information accessible to all remains a practical challenge.

### Perspectives of speakers at IPCC outreach events

The biggest challenge faced by speakers at IPCC outreach events was a lack of knowledge about the number of attendees and the composition of the audience prior to an event. This made it challenging to ‘pitch’ scientific material at the appropriate level and to tailor key messages from the IPCC reports for specific groups. For instance, at an outreach event for the SRCCL, representatives from women’s organisations responded positively to the survey questions but indicated a desire to see more discussion of gender issues in the context of climate change. All speakers agreed that their contribution to outreach events was associated with a ‘feel good’ factor. They felt their contribution made them more purposeful towards their research. However, at times, speakers felt that it was stressful when the time devoted for outreach events conflicted with their responsibilities in their day jobs. The most challenging issue for speakers, overall, was the inability to deliver the event in local languages (Table 2).

### Challenges for organizers of outreach events

#### Availability of experts in the region

Often, only a few authors from the local region were invited to IPCC outreach events, due to a limited number of regional experts and resource constraints. In such cases, organisers had to rely on non-IPCC local experts to serve on panels as moderators or speakers. Since these individuals may not have in-depth knowledge of IPCC products compared to IPCC authors, discussions at times tended to deviate away from the formal key messages of IPCC reports to more general local issues.

#### Involving local authorities and government officials

Engaging government officials as key speakers remain a challenge. Unforeseen circumstances leading to speakers cancelling the engagement create challenges in terms of securing a last-minute replacement and can leave a gap in regional expertise.

#### Time management

Ensuring key speakers stick to the allotted time can be challenging. On occasions that speakers spill over their allotted time, the time for Q&A was compromised. This indicates the importance of the role that good moderators can play.

#### Resource constraints

In case of limited availability of human resources, IPCC authors and organisers have to multi-task — from structuring events and preparing presentation content to managing social media updates. If an event is hosted at an international or national level, a professional event manager or event management team would ensure the event does not become too overwhelming or exhausting for any individual. Not all heroes wear capes: in-house support staffs are critical to successful outreach events, e.g. janitors, drivers and caterers. A simple message of appreciation following a successful event goes a long way to boosting morale and ensuring a good atmosphere for future events. Sometimes, events require support from student volunteers, and their efforts are not always fully appreciated.

Various efforts including sending group as well as personal invites, publicising events at various institutions and circulating event information in professional networks were made in an attempt to engage a diverse audience. However, factors such as an individual’s competing commitments, timing of the events, possible concurrence with other events, topic and focus of the event influence participation. Some of these aspects are beyond the organisers’ capacity to address.

#### Other factors

The indoor environment in event venues and the facilities available are important for hosting a good event. For example, at one of the events hosted in Ahmedabad during summer, the average temperature was 43 °C, and access to air-conditioned rooms was challenging. Internal lighting and acoustic conditions are also important factors in ensuring smoother interaction between the speakers and the audience. Breaks offer an informal atmosphere, which had a significant impact on interaction with IPCC authors during events. The quality of refreshments during breaks was particularly appreciated by participants.

During a roundtable event on Climate Change and Cities, an interactive exercise was developed to increase audience participation. For one event for school children, an IPCC author from the region acted as a real time translator. Access to technically enabled translation would be valuable in future when organising local events. This would, however, impose financial constraints.

### Feedback from outreach events with younger audiences

The observations in the following section are from our personal interactions with young participants at IPCC outreach events. In all, nearly 1500 students have participated in such events in India, and many of the insights resonate with the findings from the participants’ survey. For example, students appreciated the videos developed by the IPCC communications team, describing these visually attractive and interactive sessions as a pleasant change from the conventional ‘lecture model’ they are used to. While students found the outreach events inspiring in general, quite a few commented on the apathy of adults towards climate action. One student quoted “I feel very strongly about climate change, but how do I influence the adults around me who do not share the same sentiment?”.

For school students, the presentations were delivered in the form of storytelling, and these were modified depending on the age groups. At one event in Kolkata, organisers asked students to write a short paragraph on global warming in their own language and their learning from the sessions that they had attended. All responses were collected by IPCC authors, and some scanned responses were uploaded on the IPCC website. Many blogs were written in local languages expressing an appreciation for this process, which provided a great boost to students. At another event in Ahmedabad, the organisers awarded a prize for the top three responses from students on the question of ‘What can I do to address climate change?’ In Kolkata, IPCC authors acted as judges for a college students’ debate, which created extreme enthusiasm in the topic among the audience.

## Limitations and recommendations for future outreach events

The primary limitation of this study is that the survey was conducted several months after the events took place, as a result of which several participants commented on their difficulty in recalling detailed and specific feedback. This also made it challenging to secure a larger number of participant responses. This was further compounded by the changing COVID-19 situation. It is therefore strongly recommended that outreach events seek feedback during or immediately after an event. An online survey may also limit an individual’s ability to respond to certain information or provide very critical feedback, compared to a personal interview. This could potentially result in a skew towards reporting only positive experiences of the events they had attended.

Our survey participants were from urban areas of India, and, therefore, our study has not captured insights relating to communicating to people from rural areas, who are traditionally not as familiar with English. The study is limited to the events the authors of this essay hosted, attended as speakers or organised at their own institutions/through their contacts with various institutions. One of the events was a special panel in a prominent regionally hosted global conference. A future study could, therefore, be more comprehensive in terms of coverage.

In general, it is our experience that IPCC outreach events focusing on a specific theme or sector attract higher stakeholder engagement compared to more general events. A diverse set of speakers with specific individual expertise and communication skills are a key attraction for participants. Prior availability of information about audience composition could help speakers prepare in advance and communicate messages that are tailored to the specific interests and values of the group. Developing ‘event outcomes’ in the form of conference proceedings or discussion papers can become a useful resource for future events. Organisers can plan for these in advance, record feedback systematically and involve a few selected participants to contribute after the event.

Developing countries face a challenge in securing appropriate venues with good infrastructure, which our survey indicates plays an important role in the success of an event. Researchers play a “Jack of all trades” role due to limited human resource availability at local levels and, at times, play a number of these roles simultaneously, from speaker to social media manager. The limited availability of sectoral experts at a local level can perhaps be partly resolved by implementing a hybrid format that combines elements of both physical and virtual meetings with appropriate infrastructure in place.

Interview responses clearly indicate that top-down approaches to communicating climate science are not as popular or effective for engaging participants as dialogue and interaction with the speakers. Nearly all participants felt that more time should be provided for interaction. Organisers need to seek new engaging and creative formats for sessions to encourage higher participation. Coffee and tea breaks provided a useful opportunity for interaction, and these should not be compromised to adjust for time spill overs during sessions. For future events, it is recommended not to crowd events with too many speakers but leave it spaced out for better interaction, but one take home message was presenting the materials from all working groups (including aspects of physical climate science, adaptation and mitigation) makes the storyline very comprehensive and clear for the audience. Establishing methods for involving local authorities and government officials is critical for climate policy and action. More inclusive participation is also required for people who are physically challenged or who have visual and sensory impairments and needs special facilitation such as access infrastructure and sign language translator.

As mentioned earlier, the IPCC has faced challenges with regards to communicating complexity and uncertainty; therefore, precise and clear data representation and visualizations play a critical role for effective climate change communication. Together with reducing jargon and simplifying key messages, this could be achieved with the support of country-specific communications experts or local professionals if resources permit. Many participants said they only came to know through these outreach events that all IPCC reports are freely downloadable and contain an updated literature review and sections on knowledge gaps. Researchers, in particular, found this extremely useful. If outreach events are supplemented with sessions in local languages, this could allow for greater representation of local groups and individuals. Outreach for younger audiences is more challenging to organise and deliver but can be very rewarding. The new and innovative approaches to making the events more interactive were useful in attracting interest, such as designing games or debate, local participants sharing their own experiences of impacts and actions and awarding prizes. Some of the subnational governments used these collaborative events to show case their good practice in environmental and climate actions, and media coverage at regular intervals continued even weeks after the event.

This study documents, for the first time, an analysis of IPCC outreach events in India in recent years. Despite the challenges to organising and delivering IPCC outreach events in India, the events analysed here resulted in positive outcomes for speakers, organisers and participants.^13^ While this suggests these events have been successful in providing a platform for reaching a wider audience with IPCC reports, it is our hope that our recommendations will be helpful for designing and delivering outreach activities for different audiences in India for AR6 and that these insights may also be relevant for other developing countries of similar context.

## Data Availability

Not applicable.

## Footnotes

1 Quoted: “Most liked—the content and quality of the presentation and speaker's background”.

2 Quoted: “I loved how the sessions were so informative without being overwhelming…. the presenters discussed everything in a very simple, digestible manner; this made the information in the session easy to grasp.”.

3 Quoted: “That you reached to the local stakeholders to present and discuss the special reports was the most I liked about the events.”.

4 Quoted: “Most Liked—Diverse discussion that took place”.

5 Quoted: “Selection of speakers for the event was excellent which allowed to bring in diverse perspectives on the critical issue of climate change.”.

6 Quoted: “The speakers provided a detailed answer yet not using a lot of jargon so that the message was conveyed effectively to the participants.”.

7 Quoted: “The use of sustainable practices from the choice of sustainable locally sourced lunch and refreshments to the eco-friendly jute folder helps drive home the message of Practice what you preach.”.

8 Quoted: “From the events and conferences, I developed a perspective to look at the climate change from the mitigation aspects. It familiarised me with some new compelling concepts of climate change and how human behaviour, targeted scientific research, and government policies can help fight the climate crisis.”.

9 Quoted: “I was especially motivated after the event, "IPCC Dissemination Workshop: Key Messages from Three Special Reports of IPCC—14 & 15th November 2019". Interesting topics ranging from health effects of pollution in the city of Ahmedabad, change in global temperature to land use and the entire food supply chain were discussed. Answers to questions raised by the listeners were dealt appropriately.”.

10 Quoted: “I was able to openly and healthily present own thoughts on the topics of discussion, and understand how to connect with the audience effectively.”.

11 Quoted: “Least—We could have had better dialogues to produce something worthwhile from the presentations.”.

12 Quoted: “What I liked the least was the limited interaction with the audience.”.

13 Quoted: “Well, I was inspired way before such seminars to take climate actions so to me, it wasn't that inspiring, still if I want to rate, it did a small increment to 4.5 from 4.”.
